# Adaptability of Ultrasonic Lamb Wave Touchscreen to the Variations in Touch Force and Touch Area

**DOI:** 10.3390/s21051736

**Published:** 2021-03-03

**Authors:** Zengchong Yang, Xiucheng Liu, Bin Wu, Ren Liu

**Affiliations:** Faculty of Materials and Manufacturing, Beijing University of Technology, Beijing 100124, China; yzcaskj@emails.bjut.edu.cn (Z.Y.); wb@bjut.edu.cn (B.W.); 919465593@emails.bjut.edu.cn (R.L.)

**Keywords:** lamb wave, touchscreen, touch localization model, robustness

## Abstract

Previous studies on Lamb wave touchscreen (LWT) were carried out based on the assumption that the unknown touch had the consistent parameters with acoustic fingerprints in the reference database. The adaptability of LWT to the variations in touch force and touch area was investigated in this study for the first time. The automatic collection of the databases of acoustic fingerprints was realized with an experimental prototype of LWT employing three pairs of transmitter–receivers. The self-adaptive updated weight coefficient of the used transmitter–receiver pairs was employed to successfully improve the accuracy of the localization model established based on a learning method. The performance of the improved method in locating single- and two-touch actions with the reference database of different parameters was carefully evaluated. The robustness of the LWT to the variation of the touch force varied with the touch area. Moreover, it was feasible to locate touch actions of large area with reference databases of small touch areas as long as the unknown touch and the reference databases met the condition of equivalent averaged stress.

## 1. Introduction

Touchscreens have been widely used as an attractive tool to realize convenient communication between human and machines [1]. Touchscreens can be categorized based on their working mechanism as resistive-, capacitive-, optical-, and acoustic-based touchscreens. Compared with other types of touchscreens, acoustic touchscreens have two significant advantages. First, the touch action-induced localized pressure disturbs the acoustic field in the media of screen. Through analyzing the disturbed acoustic field, the acoustic touchscreen is capable of recognizing both the location and contact pressure of a touch action [2,3], thus enriching the functions of the touchscreen without additional force sensors. Second, the acoustic field interacting with the touch action can be constructed in elastic solid media, including but not limited to transparent glass. Unimpressive things such as a metal shell of robot arm, wood or plastic desk might be turned into tactile sensing media based on acoustic touchscreen technology in the future.

Acoustic touchscreens utilize surface acoustic wave (SAW) [2,4] or ultrasonic Lamb wave to interact with the touch action. The energy of the SAW travels at the near surface of a screen and thus the SAW touchscreen is sensitive to contaminants (such as sweat and dew) and scratches. Contaminants and scratches may cause the recognition failure of touch actions. Compared with the SAW, the Lamb waves of the selected mode and operation frequency propagate through the entire cross-section of a plate screen. Therefore, in recent years, the Lamb wave touchscreen (LWT) has been developed to overcome the deficiency of SAW touchscreen. The LWT works in passive [5,6,7,8,9,10,11,12] and active modes [3,13,14,15,16,17,18,19,20,21,22,23,24]. In the former mode, the Lamb wave is generated by the touch action and received by the piezoelectric wafer attached to the edge of the plate. However, the holding action as a static load does not generate Lamb wave and cannot be recognized by the passive LWT. In the active mode, the piezoelectric wafer can be employed for generating and receiving Lamb wave. Any static or transient load caused by touch action disturbs the field of Lamb wave and can be identified by the active LWT.

In the development of LWT, the localization of a touch action is firstly concerned. The developed touch action localization methods for LWT can be simply classified into unsupervised and supervised algorithms. In the unsupervised algorithms, the location of an unknown touch is directly calculated with the time delay of arrival (TDOA) technique [5,6,7,8] or its variants [23]. Advanced training with a database of acoustic fingerprints of touch action is commonly carried out with supervised algorithms. During the derivation of database, the sensing region of the touchscreen is meshed into certain pixels. Touch action is then gradually applied at each pixel. The response signals of touch (referred to as acoustic fingerprints) at all the pixels are recorded as a database. The acoustic fingerprints with prior knowledge on their touch positions are directly used as the reference signal database [3,9,10,11,12,13,14,15,16,17,18,20,21,22] or used to establish a locating model with neural network [24]. To achieve accurate localization of the unknown touch with the responded Lamb waves, various types of methods such as amplitude disturbed diffraction pattern [14,15,16], contact impedance mapping method [3,18], and learning method [20,21,22] have been explored. However, in previous studies, an unknown touch was localized with the database established with the same touch (the same touch force and touch area). The effect of touch force or touch area on the performance of localization methods for LWT has not been explored.

In this study, LWT employing a Corning glass plate was constructed in our laboratory. Multiple databases of acoustic fingerprints were acquired under touch actions of different forces and areas. An improved learning method with self-adaptive weights was used to improve the accuracy of touch action localization. Then the compatibility of rigid database and the robustness of the improved learning method to the varying touch force and touch area were investigated. The rest of the paper is organized as follows. The experimental system and scheme for automatic acquisition of acoustic fingerprint of touches are introduced in Section 2. The improvement in the learning method using self-adaptive weight is described in Section 3. The effects of touch force and area on the performance of Lamb wave touchscreen in touch localization are discussed in Section 4. Finally, the conclusions are drawn in Section 5.

## 2. LWT Platform and Database Collection

**Figure 1:**
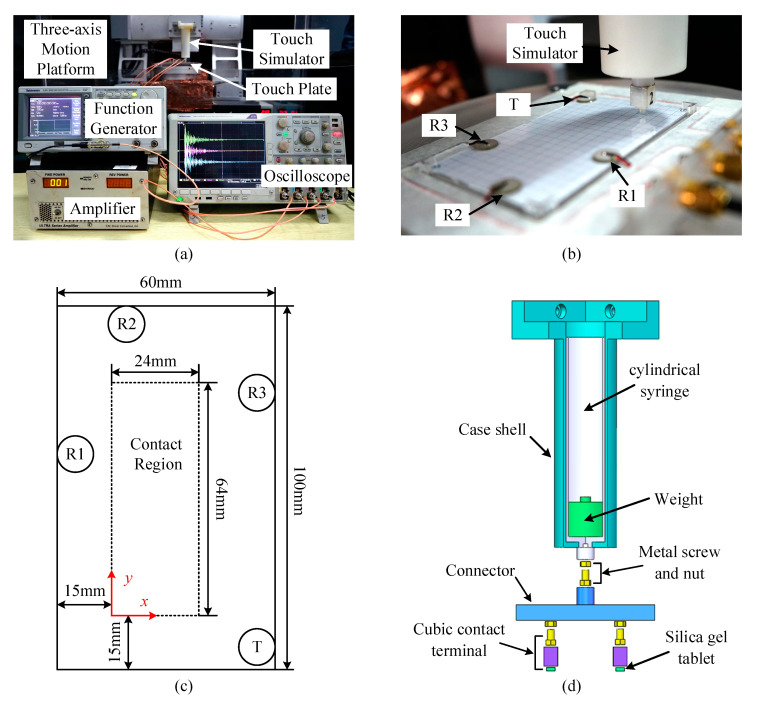
Experimental system for LWT verification: (**a**) picture of entire system; (**b**) picture and (**c**) diagram of the sensor layout on the plate; (**d**) configuration of the touch simulator.

The experimental system for LWT verification (Figure 1a) was established in our laboratory. Disk-shaped PZT-5H transducers with a diameter of 10 mm and a thickness of 1 mm were glued on a Corning glass plate (as touchscreen) with a size of 100 × 60 × 0.8 mm^3^ by using the Devcon industrial epoxy adhesive (Model: 14250). A square pulse with a pulse width of 5 μs was generated by Tektronix function generator (Model: AFG 3021B) and amplified by a power amplifier (Brands: T&C Power Conversion, Inc., New York, NY, USA, Model: AG 1006) before it was used to actuate the PZT-5H transducer. Previous studies emphasized that asymmetric placement of sensors could reduce the identification error caused by mirrored touch positions [16]. Therefore, the Lamb wave transmitter was attached at a corner of the plateand three receivers were attached at non-mirrored locations at different side edges of the touchscreen (Figure 1b,c). The Lamb waves propagating through different paths were received by the three receivers and synchronously acquired by a Tektronix digital oscilloscope (Model: DPO 4054B) with a sampling frequency of 5 MHz.

To simulate the touch action, a touch simulator (or artificial finger) with the configuration sketched in Figure 1d was designed. A connector, a cylindrical syringe, and a case shell were fabricated by 3D printing technique with resin. A metal screw and a nut were embedded into the connector and the syringe needle, respectively. Cubic contact terminals of resin were prepared with 3D printing technique and connected to the connector with the embedded screw and nut. Multi-touch actions could be simulated with several cubic contact terminals connected to the connector. Figure 1d shows a simulator of two touches. One touch could be realized by directly connecting cubic contact terminals to the syringe needle. After the connector or the contact terminal was connected with the syringe, different weights were loaded on the end of the syringe. To simulate the finger skin, a square piece of silica gel was attached onto the bottom of the contact terminal. Through replacing the weight and the contact terminal with silica gel tablets of different sizes, the touch force and the area could be changed.

**Figure 2:**
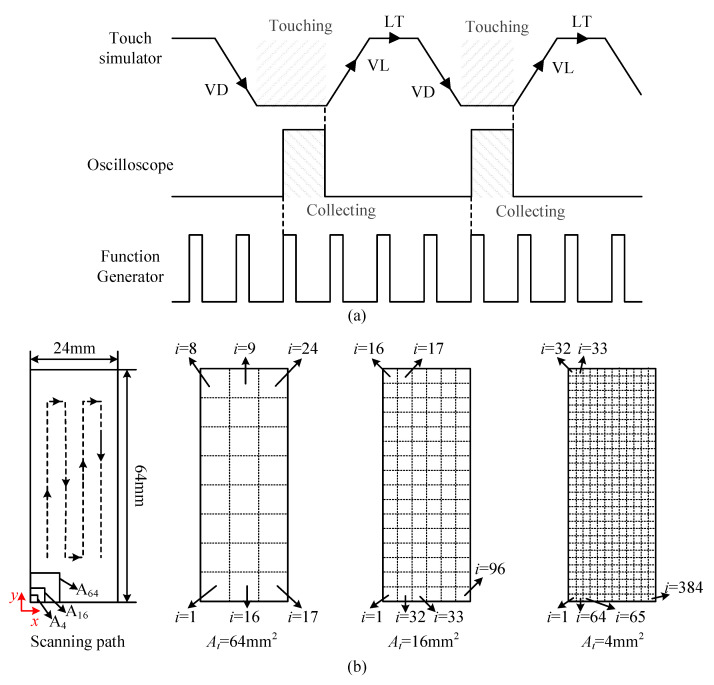
Automatic data acquisition scheme: (**a**) sequence diagram of the system and (**b**) pattern of pixel division and scanning path of the touch simulator.

The touch simulator was installed onto the beam of Z-axis of a three-axis motion platform. The operation sequence of the motion platform and the Lamb wave inspection devices were controlled by the LabVIEW program run in a host computer. To realize the automatic collection of the acoustic fingerprints of touch actions, the operation of the entire system is performed according to the sequence diagram shown in Figure 2a. During the process of database collection, the function generator continuously output a sequence of square pulses with a duty cycle of 50%. The periodical motion of the touch simulator could be divided into four stages: vertical descending (VD), touching, vertical lifting (VL), and lateral translation (LT). At the end of the vertical descending stage, the digital oscilloscope was triggered to wait for the next pulse excitation and the data acquisition started after the arrival of the rising edge of the excitation pulse. When the data acquisition was completed, the motion platform was triggered to carry the touch simulator away from the screen. The Supplementary Video S1 displayed the data acquisition process in an experiment.

The contact region (64 × 24 mm^2^) shown in Figure 1c was evenly divided into square grids (or pixels). Three cases of single touch with pixels of 4, 16, and 64 mm^2^ (referred as the touch area *A_t_*) were investigated in the database collection. The pixel at the low left corner was labeled as the first touch position (*i* = 1) for the acoustic fingerprint collection. The order of the *i*-th touched pixel is consistent with the scanning path of the touch simulator shown in Figure 2b. For the convenience of understanding, the position of the pixel could be defined with its row and column coordinates in the segmented contact region. For instance, the position of the fifth touched pixel can be defined with *x* = 1 and *y* = 5. Once the touch area and force were fixed, the point-by-point touch action was applied in the contact region and the database of acoustic fingerprints of touch actions were automatically collected to suppress the uncertainty and save time. When the touch area was selected as *A_t_* = 4 mm^2^, *A_t_* = 16 mm^2^, and *A_t_* = 64 mm^2^, the contact region was evenly divided into 24, 96, and 384 pixels, respectively. As a result, a total of 24, 96, and 384 acoustic fingerprints were, respectively, collected in the database labelled by the touch areas of *A_t_* = 4 mm^2^, *A_t_* = 16 mm^2^, and *A_t_* = 64 mm^2^. In each case of touch area, the touch force, *F_t_*, applied by the touch simulator was alternatively refreshed in the range of 0.4~2 N with an increment of 0.4 N, and the acoustic fingerprint collection was repeated to generate new databases. The above database collection procedure was repeated five times to generate five parallel databases, and a random error was generated in the collection procedure. Finally, a total of = 75 databases of acoustic fingerprints (3 (touch area) × 5 (touch force) × 5 (times)) were collected in order to investigate the effects of touch force and area on the touch action localization performance of LWT.

**Figure 3:**
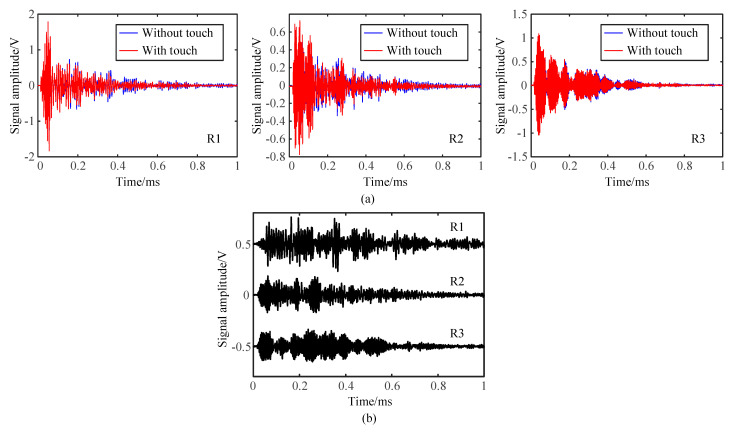
Typical acoustic fingerprints: (**a**) typical signals received with and without touch action and (**b**) disturbances of acoustic fingerprint caused by the touch action.

When a touch action and no touch action was applied in the contact region, the waveforms of the Lamb waves received by all the three receivers were plotted in Figure 3a. The differences in both propagation path and boundary reflection condition caused the diversity of Lamb waves detected by different pairs of transmitter–receivers. The minor disturbance caused by the touch action can be observed.

As an example to illustrate the acoustic fingerprints in the database, the signals collected at the pixel of (*x* = 2, *y* = 4) under the conditions of the touch simulator (a square area of 64 mm^2^ and a weight of 2 N) were recalled. To highlight the change of waveforms caused by the touch action, the signal obtained with the touch action was subtracted from the reference signal collected without the touch action. The subtraction results (Figure 3b) clearly demonstrated that the touch object caused weak and complicated disturbances to the Lamb waves propagating in the glass screen. Thus, the signals detected by all the three pairs of transmitter–receivers could be used as the acoustic fingerprint of the touch action.

## 3. Touch Action Localization Method

Though the Lamb waves received by each receiver can act as acoustic fingerprint of touch action, it is not easy to decode the exact relationship between the locations of the touch simulator with the acoustic fingerprints due to the complicated scattering of multiple modes at the touch position. In this study, the localization model of learning method [20,21,22] was improved and employed. The measured data in the presence of unknown touch(s) were considered as a linear combination of a set of collected acoustic fingerprints of touch actions in the reference database. Therefore, the signal of unknown touch, $\bar{d}$, can be expressed as:

(1) $$\bar{d}\approx \sum_{i=1}^{N_{p}}\theta_{i}d_{i},$$

where **d***_i_* denotes the reference acoustic fingerprint of the *i*-th touch pixel; *N_p_* represents the number of collected acoustic fingerprints of different touch actions; *θ_i_* is the coefficient of **d***_i_*. The localization of the touch action can be transformed into the process of solving a least-squares problem as follows:

(2) $$\underset{\Theta \in R^{N}}{\min}\frac{1}{2}{\left\|D\Theta -\bar{d}\right\|}_{2}^{2},$$

where $D=\{d_{i}{\}}_{i=1}^{N_{p}}$ is the collected database of the acoustic fingerprints of all the pixels in the contact region in the touchscreen; $\Theta =\{\theta_{i}{\}}_{i=1}^{N_{p}}$ indicates the possible position of the touch action and belongs to the dataset of real number, R*^N^*. The utilization of multiple pairs of transmitter–receivers could improve the touch object localization accuracy [22]. In the study, a total of three pairs of transmitter–receivers were used. Hence, Equation (2) can be extended to the case with one transmitter and three receivers as follows:

(3) $$\;\underset{\Theta \in R^{N}}{\min}\frac{1}{2}\sum_{r=1}^{3}w_{r}{\left\|D_{r}\Theta -{\bar{d}}_{r}\right\|}_{2}^{2},$$

where *w_r_* is the weight coefficient, and the subscript *r* represents the number of the transmitter–receiver pairs. Considering the sparse distribution of the touch objects in the positively definite and stable system, the following constraints was assigned to Equation (3),

(4) $$\theta_{i}\geq 0\;(\mathrm{for}\;\mathrm{all}\;i),\;\mu \sum_{i=1}^{N_{p}}\theta_{i}=1,$$

where *μ* is a penalty coefficient. Equations (3) and (4) constitute a complete locating or projection scheme. The locating scheme could be improved by reformulating the problem in the image space, which is spanned by all possible configurations of Θ. Two steps were required for the application of the touch object location algorithm:

Step 1: Equation (2) without constraints is solved by using the signal received by an individual pair of transmitter–receivers in order to provide the solution of touch action’s location ($\Theta_{r}^{*}$),

(5) $$\Theta_{r}^{*}=\underset{\Theta_{r}\in R^{N}}{\arg\min}\frac{1}{2}{\left\|D_{r}\Theta_{r}-{\bar{d}}_{r}\right\|}_{2}^{2},$$

Step 2: The constrained least squares problem is solved as:

(6) $$\underset{\Theta \in R^{N}}{\min}\frac{1}{2}\sum_{r=1}^{3}w_{r}{\left\|\Theta -\Theta_{r}^{*}\right\|}_{2}^{2},$$

which is subjected to,

(7) $$\theta_{i}\geq 0,\;\mathrm{for}\;\mathrm{all}\;i,$$

(8) $$\mu \sum_{i=1}^{N_{p}}\theta_{i}=\sum_{r=1}^{3}\sum_{j=1}^{N_{p}}\theta_{rj}^{*},$$

**Figure 4:**
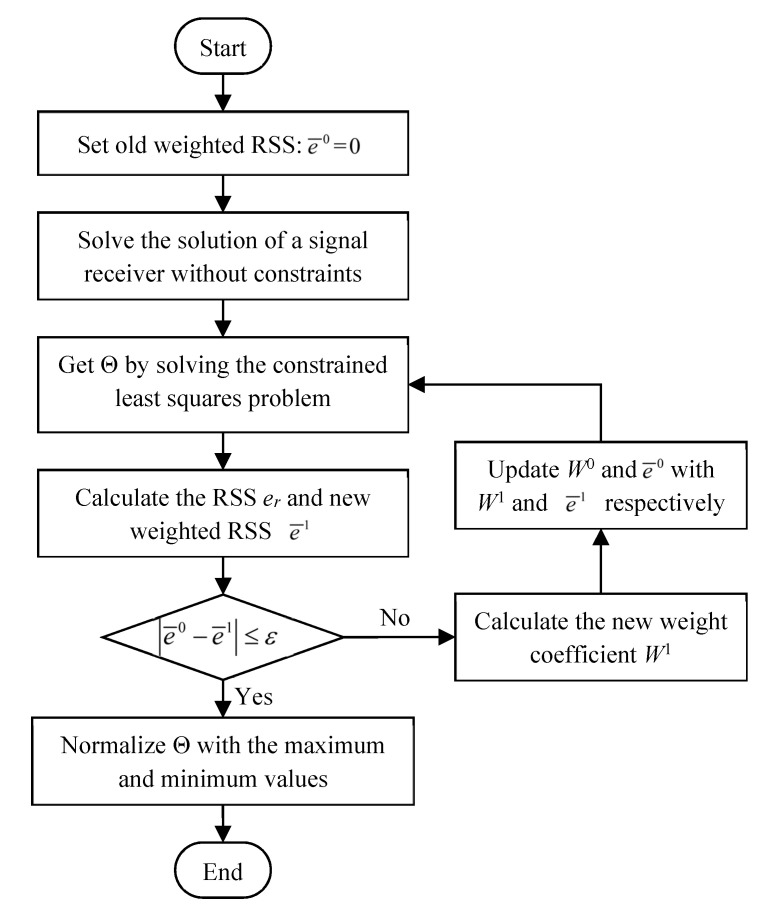
Flowchart of the improved learning method with self-adaptive weights for touch localization.

The least square problem as stated in Equation (6) can be transformed into a quadratic programming problem [25],

(9) $$\underset{\Theta \in R^{N}}{\min}\frac{1}{2}\Theta^{T}H\Theta +f^{T}\Theta ,$$

where $f=-\sum_{r=1}^{3}w_{r}\Theta_{r}^{*}$, $H=I\sum_{r=1}^{3}w_{r}$ and *I* is the identity matrix whose order is equal to *N_p_* (the number of elements in Θ). The subscript *T* denotes the operation of transposition of matrix. The problem defined by Equation (9) can be solved with the quadratic programming solver run in MATLAB platform. The weight coefficient *w_r_* in Equation (6) greatly affects the localization result of an unknown touch. In the previously reported algorithm [20,22], the criterion for selecting the weight coefficient was not discussed. The random selection of the weight coefficient may lead to the locating error of a touch action. To solve this problem, an iterative self-adaptive method was proposed in this paper based on the random weighting method proposed for multisensor data fusion [26]. The flowchart of weight coefficient optimization and touch localization is shown in Figure 4.

In this method, the residual sum of squares (RSS) is used to update the weight coefficient. The initial locating results: Θ^0^ is obtained by solving Equations (6)–(8) with the initialized weight coefficient $W^{0}=\{w_{1}^{0},w_{2}^{0},w_{3}^{0}\}$, where $\sum_{r=1}^{3}w_{r}^{0}=1$. The value of RSS (represented by the parameter of *e_r_*) for each transducer is calculated as:

(10) $$e_{r}={\left\|D_{r}\Theta^{0}-{\bar{d}}_{r}\right\|}_{2}^{2},$$

As a result, the new weight coefficient of $w_{r}^{1}$ is updated as,

(11) $$w_{r}^{1}=\frac{1}{e_{r}}/\sum_{r=1}^{3}\frac{1}{e_{r}},$$

Equations (6)–(8) is then solved with the updated weight coefficient. A weighted RSS of $\bar{e}$, which can be computed with Equation (12), is used to terminate and record the iterative process,

(12) $$\bar{e}=\sum_{r=1}^{3}w_{r}e_{r},$$

The above iterative process will not stop until the difference of the weighted RSS $\bar{e}$ between two adjacent iterations is equal to or less than a threshold *ε*.

**Figure 5:**
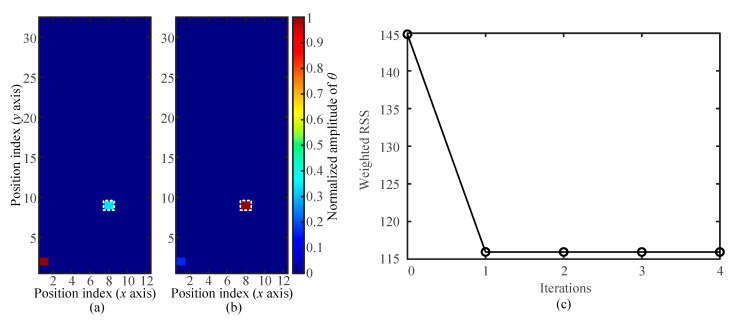
Performance comparison between the conventional and updated strategies in an unknown touch location: (**a**) and (**b**) touch location results, respectively, obtained with randomly and self-adaptively selected weight coefficients and (**c**) the value of weighted RSS in four iteration steps.

A database collected under the condition of a touch area of *A_t_* = 4 mm^2^ and a touch force of *F_t_* = 0.4 N was employed to testify the better localization performance of the updated strategy in an unknown touch than that of the conventional method. The position of the touch to be located is marked as the white dotted pixel (*x* = 8, *y* = 9) shown in Figure 5a. When the weight coefficients for the three receivers were close to each other and fixed as *W* = {0.33, 0.33, 0.34}, the solution of Equations (6)–(8) indicates that the touch action is at the pixel with the position index of *x* = 1 and *y* = 2. Obviously, the conventional algorithm outputs a wrong locating result due to the improper generation of weight coefficients. The proposed iterative self-adapting method could update the weight coefficients until the reference signals approached the optimal combination. Figure 5c shows the weighted RSS of different iteration steps in the tested case with assigned four iteration steps. The optimal weight coefficients could be obtained as *W* = {0.17, 0.56, 0.27} after only one iteration. With the optimal weight coefficients, the updated algorithm could accurately locate the unknown touch (Figure 5b).

## 4. Results and Discussion

The LWT is a database-based technology. The adaptability of the collected databases (labelled by the touch force and touch area) to an unknown touch is mainly concerned in the application. The touch area and the touch force applied to the LWT are different among individuals. Though the parameters of the touch simulator are adjustable, the establishment of databases covering all the combinations of various parameters is time-consuming and not conducive to the rapid touch localization. In the evaluation of the adaptability of the collected databases to an unknown touch, the effects of touch force and touch area on the performance of LWT in touch localization were investigated firstly in this study. The touch actions with the parameters that were different from those in the labelled database were used to evaluate the adaptability of the collected databases to an unknown touch.

### 4.1. Touch Force

**Figure 6:**
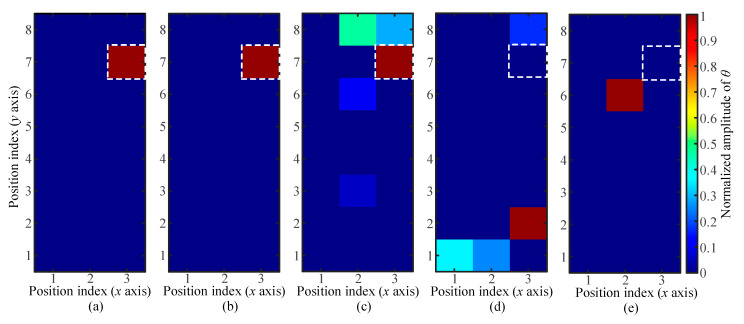
Adaptability of the LWT to an unknown touch of different touch forces: (**a**–**e**) localization results of a touch action at the pixel (*x* = 3, *y* = 7) with a touch force of *F_t_* = 2 N, *F_t_* = 1.6 N, *F_t_* = 1.2 N, *F_t_* = 0.8 N, and *F_t_* = 0.4 N, respectively.

The databases collected under the condition of a touch area of *A_t_* = 64 mm^2^ were used to demonstrate the evaluation process of LWT performance. The touch to be located was applied at the pixel with the position index of *x* = 3 and *y* = 7. This nominally unknown touch (NUT) had a touch area of *A_t_* = 64 mm^2^ and a touch force of *F_t_* = 2 N. According to the touch location algorithm shown in Figure 4, the Lamb wave signals caused by this NUT were compared with the acoustic fingerprints in the collected database labelled by the touch area (fixed as *A_t_* = 64 mm^2^) and touch force (0.4 to 2 N). During the operation of the algorithm, the value of the penalty parameter is *μ =* 1. The location results are shown in Figure 6, and the color maps are labelled by the normalized amplitude of *θ*.

Among the pixels in the color map, the pixel with the highest value of *θ* indicates the location of NUT. As shown in Figure 6, when the touch force of the NUT is equal to the touch force of the labelled database, only one pixel is highlighted, and its location is consistent with that of the NUT. The accurate location of the NUT (with touch force of *F_t_* = 2N) could also be achieved by using the database labelled with a touch force of *F_t_* = 1.6 N. In the database labelled with a touch force of *F_t_* = 1.2 N, though multiple pixels were highlighted as possible locations of the NUT, the pixel with the highest value of *θ* indicated the actual location of the NUT. Therefore, the slight mismatch of touch force between the database’s label and the NUT did not lead to location error. In other words, the collected databases together with the updated location algorithm had certain robustness to the varying touch force of an unknown touch. However, location errors occurred when the databases of the touch forces of *F_t_* = 0.8 N and *F_t_* = 0.4 N were used for NUT location (Figure 6d,e).

**Figure 7:**
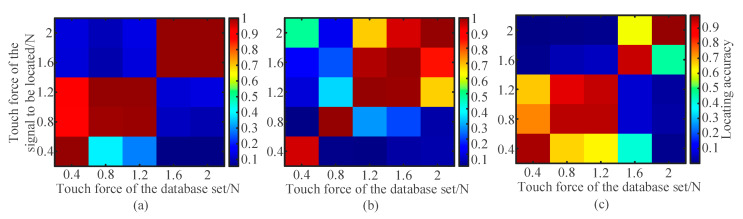
Cross-validation results indicating the tolerance zone of LWT to the variation in touch force. (**a**–**c**) Cross-validation results obtained with the databases with labelled touch area of *A_t_* = 64 mm^2^, *A_t_* = 16 mm^2^, and *A_t_* = 4 mm^2^, respectively.

To comprehensively evaluate the adaptability of the collected databases to the varying touch force of an unknown touch, cross-validation was performed with the collected databases labelled with the same touch area. As mentioned previously, five cases of different touch forces were tested with the touch simulator of a given touch area. As each case (labelled as *F_t_* and *A_t_*) of touch force was repeated five times, a total of 25 databases (5 (touch forces) × 5 (times)) were collected. Each of the 25 databases alternatively acted as the reference database for an unknown touch location. All the acoustic fingerprints in the 25 databases were treated as the signal caused by an unknown touch. As a result, a cross-validation matrix of 25 × 25 was generated. At each element in the matrix, the unknown touch location was executed for *N*_p_ times. The location number of the unknown touches with the same touch force was *N*_p_ × 25. Through averaging the location accuracy of all the unknown touches with the same touch force, the cross-validation matrix could be transformed into be a location accuracy matrix with a dimension of 5 × 5 (Figure 7).

The consistency between the parameters (touch area and touch force) of the unknown touch and the database’s label could ensure that the unknown touch could be accurately localized with a maximum probability. Therefore, in the maps shown in Figure 7, the pixels at the diagonal line crossing zero point have the highest localization accuracy. The mismatch between the touch forces of the unknown touch and the database caused the localization error or the decreased localization accuracy of LWT. The pixels at the upper left and lower right corners were located at the region with the most mismatched touch force, indicating the localization failure in an unknown touch.

The localization accuracy was high in the upper right and lower left zones relative to the diagonal line, so these zones are referred to as tolerant zones. For instance, as indicated in Figure 7a, the reference databases labelled with a touch force ranging from 0.4 N to 1.2 N can accurately localize the position of an unknown touch with a touch force ranging from 0.8 to 1.2 N. Similar conclusions can be respectively drawn from Figure 7b,c under the touch forces of 1.2~2 and 0.4~1.2 N. In the tolerant zones, the mismatch between the touch forces of the unknown touch and the database’s label had a limited effect on the localization accuracy, thus confirming the robustness of the LWT to the varying touch force. It is worth noting that the touch force tolerant zone varies with the touch area. The tolerance of LWT to the variation of touch force could be ascribed to the highly similar acoustic fingerprints of the touches with different touch forces. The touch action-induced disturbance to the Lamb wave field was related to both the touch force and the area. It is recommended to use the localization accuracy map shown in Figure 7 to calibrate the touch force tolerant zone according to the required pixel resolution.

### 4.2. Touch Area

**Figure 8:**
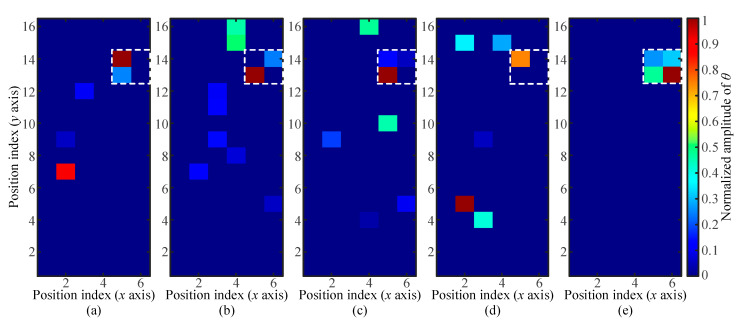
Localization results of a touch action of *A_t_* = 64 mm^2^ and *F_t_* = 1.6 N with the database labelled as *A_t_* = 16 mm^2^. (**a**–**e**) Localization results, respectively, obtained with the reference databases of *F_t_* = 2 N, *F_t_* = 1.6 N, *F_t_* = 1.2 N, *F_t_* = 0.8 N, and *F_t_* = 0.4 N.

The contact area between a human finger and the screen is around 1 cm^2^. The touch areas of the acoustic fingerprints in the collected database are smaller than that of a touch action implemented by a human finger. In this study, only the feasibility of the application of the databases obtained with small touch areas in localizing the unknown touch with a large touch area was investigated. First, the acoustic fingerprint with a touch area of *A_t_* = 64 mm^2^ in and a touch force of *F_t_* = 1.6 N was used as a nominal unknown touch, which was then localized with the reference databases obtained with a touch area of *A_t_* = 16 mm^2^. A NUT with an area of *A_t_* = 64 mm^2^ covered four pixels of *A_t_* = 16 mm^2^. The NUT localization results obtained with different reference databases and the actual touch location (white dotted pixel) are shown in Figure 8.

The localization of NUT would be considered to be successful if the pixel with a maximum value of normalized *θ* was in the region of actual touch location. In the tested four cases (with different touch forces), only the results in Figure 8d failed to locate the real touch position. The touch action applied onto a glass screen changed the boundary conditions of the wave motion equation. Though the exact mechanism of the localized load affecting the propagation of Lamb waves was not revealed, the stress field applied to the contacted surface of the screen was the main factor determining the feature of acoustic fingerprints. When the touch simulator applied a load of *F_t_* = 1.6 N in the area of *A_t_* = 64 mm^2^, an averaged stress of *σ*_a_ = 25 kPa was imposed onto the contact area. The averaged stress corresponding to the five reference databases used in Figure 8 ranged from *σ*_a_ = 25 kPa to *σ*_a_ = 125 kPa. The tolerance of LWT to the varying touch force had been proven in Section 4.1. As observed from Figure 8a–c, with the robust locating algorithm, the touch stresses of *σ*_a_ = 125 kPa, *σ*_a_ = 100 kPa, and *σ*_a_ = 75 kPa can be successfully located though randomly distributed noised pixels contained in the maps. It is surprising that the touch stress of *σ*_a_ = 25 kPa is perfectly localized. No noised pixel is found in Figure 8e.

**Figure 9:**
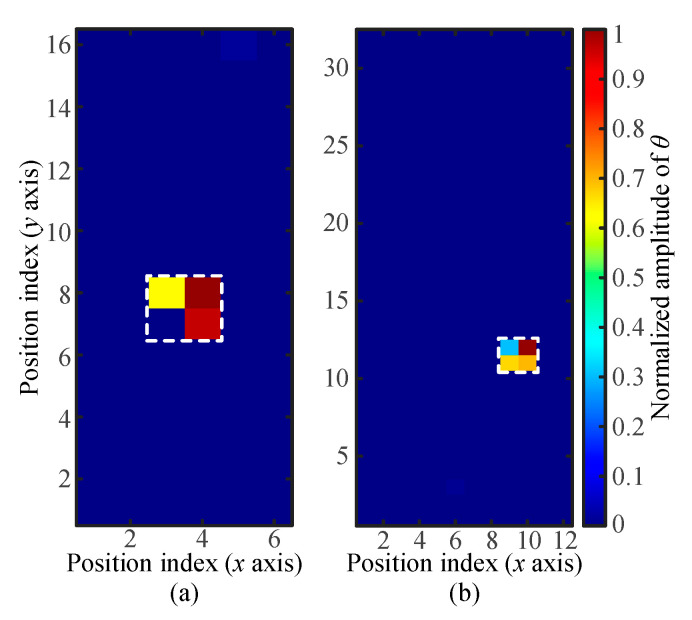
Localization results of a large-area touch with the reference database of a small area under the equivalent condition of averaged stress: (**a**) Localization results of unknown touch and the reference database with the *A_t_* combination of 64 and 16 mm^2^ and (**b**) localization results of unknown touch and the reference database with the *A_t_* combination of 16 and 4 mm^2^.

When the NUT with a touch stress of *σ*_a_ = 25 kPa moved to another position, accurate touch localization could still be realized (Figure 9a). The reason might be interpreted as follows. The averaged touch stress of the NUT was equal to that of the reference database, so the acoustic fingerprint of a large-area touch could be considered as the linear superposition of acoustic fingerprints of small-area touches. The linear superposition effect was consistent with the assumption stated in Equation (1). Therefore, when the database obtained with small touch areas was used to locate a NUT with a large touch area, the highest localization accuracy was obtained under the condition that the averaged stresses of the NUT and the reference database were the same. Another localization case was investigated after the touch areas of NUT and the reference database were, respectively, changed into 16 and 4 mm^2^ under the fixed averaged stress of 100 kPa. The NUT localization results in Figure 9b confirmed the conclusions obtained from Figure 8e. It is inferred that the averaged stress rather than the touch area or the touch force is the key factor in labelling the acoustic fingerprint databases.

**Figure 10:**
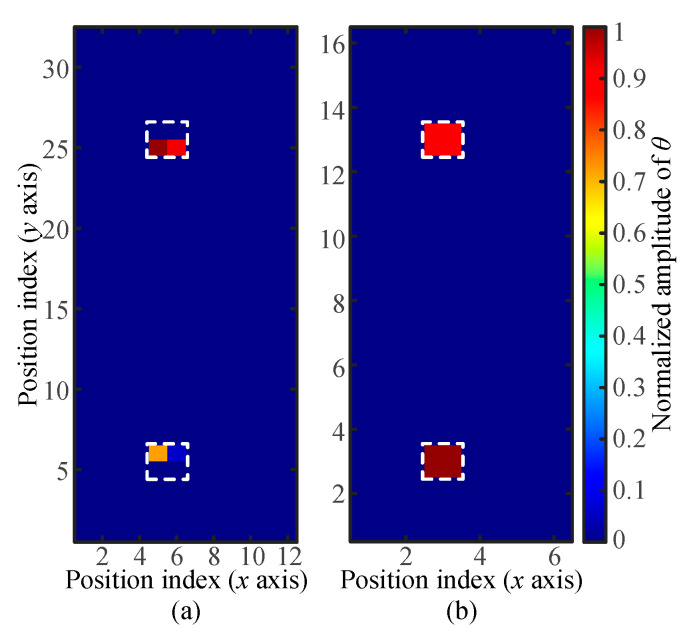
Localization results of two touches. (**a**,**b**) localization results, respectively, obtained with the reference database of *A_t_* = 4 mm^2^ and *σ_a_* = 100 kPa and the reference database of *A_t_* = 16 mm^2^ and *σ_a_* = 50 kPa.

The equivalent conditions of the averaged stress between the unknown touch and the reference databases were conducive to the localization of touch actions of a large area with the reference databases obtained with small touch areas (Figure 8 and Figure 9). The performance of LWT in locating two touches satisfying the equivalent conditions of the averaged stress was further investigated. A touch simulator with two identical cubic contact terminals of *A_t_* = 16 mm^2^ in the touch area acted as the unknown touch. The load applied by the touch simulator was around *F_t_* = 1.6 N, and each of the cubic contact terminals applied an averaged stress of *σ*_a_ = 50 kPa onto the glass screen. First, the reference database (*A_t_* = 4 mm^2^, *F_t_* = 0.4 N, *σ*_a_ = 100 kPa) was used to localize the two identical touches with an averaged stress of *σ*_a_ = 50 kPa, which was different from that in the reference databases (100 kPa). The located pixels were in the actual contact region (Figure 10b). However, the number of the highlighted pixels was not consistent with the actual situation of four adjacent pixels, and the difference between the values of *θ* for the two highlighted touches was significant. Second, the reference database (*A_t_* = 16 mm^2^, *F_t_* = 0.8 N, *σ*_a_ = 50 kPa) was used for unknown touch location. The localization results shown in Figure 10a verified that the synchronous and accurate location of two touches could be realized. The comparison between the results in Figure 10a,b proved that the unsatisfied equivalent condition of averaged stress led to the insufficient localization accuracy of two identical touches. 

## 5. Conclusions

An experimental prototype of LWT employing one transmitter and three receivers was established in our laboratory. The automatic collection of acoustic fingerprints of touch actions was realized by adjusting the operation sequence of the touch simulator and Lamb wave inspection devices. Through changing the area of the silica gel tablet on cubic contact terminals and the weight in a cylindrical syringe, touch simulators with different parameters were prepared. With the proposed experimental prototype of LWT, the databases of acoustic fingerprints under different touch forces and touch areas were successfully collected. The performance comparison between the conventional and updated strategies in unknown touch location indicated that the localization model of learning method with self-adaptively selected weight coefficients had higher accuracy than the model based on randomly selected weight coefficients. With the improved unknown touch location algorithm, cross-validation was performed among the databases labelled by different touch forces and touch areas. The LWT had a certain degree of adaptability to the variations in touch force and touch area. Importantly, an equivalent condition of averaged stress between the unknown touch and the reference databases could be used to accurately locate single- and two-touch actions of a large area with reference databases of small touch areas. In the future, the improved learning method will be used to localize multi-touch actions comprehensively and identify the touch force.

## Supplementary Materials

The following are available online at https://www.mdpi.com/1424-8220/21/5/1736/s1, Video S1: Automatic data acquisition experiment of Lamb wave-based touchscreen.
